# Characterization of Machined Surface Topography Based on the Normal Declination Angle of Microfacets

**DOI:** 10.3390/mi12030228

**Published:** 2021-02-24

**Authors:** Wei-Chao Shi, Jian-Ming Zheng, Qi-Long Wang, Li-Jie Wang, Qi Li

**Affiliations:** School of Mechanical and Precision Instrument Engineering, Xi’an University of Technology, Xi’an 710048, China; zjm@xaut.edu.cn (J.-M.Z.); wangql@xaut.edu.cn (Q.-L.W.); wanglj@xaut.edu.cn (L.-J.W.); liqi@xaut.edu.cn (Q.L.)

**Keywords:** characterization method, microfacet theory, normal declination angle, machined surface topography, lognormal distribution

## Abstract

It is important to characterize surface topography in order to study machined surface characteristics. Due to the features of periodicity and randomness of machined surface topography, the existing topographical parameters may not describe its features accurately. A novel characterization method called the normal declination angle of microfacet-based surface topography is thus proposed for this task. The topography of machined surfaces is measured and the data on the normal declination angle are obtained. Then, surface topography is analyzed via the distribution of the normal declination angle. The lognormal distribution characterization model of machined surface topography is established, and the accuracy of the model is verified by error analysis. The results show that the calculated results of the present characterization model are generally consistent with the distribution of the normal declination angle, where the maximal root mean square errors (RMSE) is 4.5%. Therefore, this study may serve as an effective and novel way to describe the characteristics of the machined surface topography.

## 1. Introduction

Machined surface topography refers to the microgeometry shape left on a workpiece surface by tools and chips during the machining process [1], which is widely used in the research fields of joint surface characteristics, lubrication part design, surface scattering characteristics, and 3D reconstruction [2,3,4]. Therefore, it is of great significance for modern engineering to accurately and effectively characterize machined surface topography [5,6].

The characterization methods of surface topography mainly include the topographical parameter method [7], fractal method [8], wavelet method [9], and autocorrelation function method [10], among which the topographical parameter method is considered to be one of the fastest and most efficient characterization methods [11,12,13]. Therefore, the characterization method of topographical parameters has attracted attention from numerous researchers. Panda et al. studied the effect of the topography of counter-surfaces on the wear of engineering polymers by using the topographical parameters, and established the characterization model of counter-surfaces topography based on the Weibull distribution, realizing the prediction of counter-surfaces on the wear of polymers [14]. Pawlus et al. proposed the use of two parameters to characterize the shape of the ordinate distribution of a two-process profile, which analyzed the statistical dependencies between various non-dimensional parameters on the basis of the linear coefficient correlation. This process provided an accurate description of the ordinate distribution of the two-process profile by using the ratio of *R_p_*/*R_z_* and *R_q_*/*R_a_* [15]. Krolczyk et al. studied surface topography after turning in the dry and minimum quantity cooling lubrication (MQCL) conditions using the parametric and nonparametric method. The surface amplitude parameter and contour map were used to describe the characteristics of the surface topography because they realized the comparative analysis of the surface topography under different machine conditions [16]. The existing topographical parameters are mainly used to describe distribution heights of the surface topography. However, machined surface topography belongs to a non-smooth and discontinuous surface and has the features of periodicity and randomness. Therefore, the existing topographical parameters make accurately characterizing the machined surface topography difficult.

The study of the microfacet theory began in the 1960s. Torrance et al. proposed the hypothesis that an object’s surface was composed of numerous microfacets for the characterization of a rough surface, which laid the foundation for the microfacet theory [17]. Yang et al. proposed a modified microfacet polarized bidirectional reflectance distribution function (pBRDF) model by combining the microfacet and Kubelka–Munk theories, both of which expressed the simulation of the degree of polarization characteristics for material surface in the pBRDF model [18,19]. Meneses et al. presented a non-contact methodology for the in situ determination of both the temperature and spectral emissivity of a surface spot based on the microfacet theory. This established the mapping relationship between surface light scattering characteristics and temperature, which expressed the determination of the surface temperature [20].

For the aforementioned problems, this study proposes a new surface topography characterization method based on the microfacet’s normal declination angle. By combining the principle of the microfacet and the mathematical statistics method, different machined surface topographies are analyzed, and the characterization model of the machined surface topography is established. This paper provides an effective and novel characterization method for surface topography.

## 2. The Characterization Method for Surface Topography

**Figure 1:**
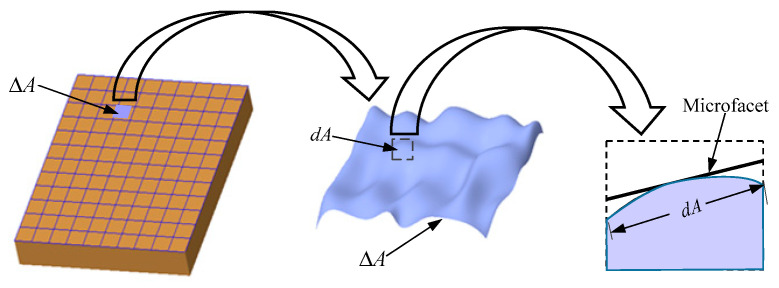
Microfacet model.

According to the microfacet theory, any local area Δ*A* of an object’s surface is composed of numerous topographies *dA*, wherein the area of the topography *dA* is small. Therefore, the tangent plane of the topography can be used to describe the topography, i.e., the microfacet, as shown in Figure 1.

**Figure 2:**
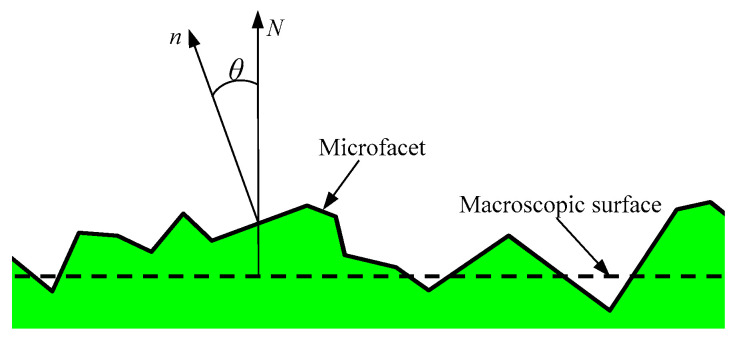
Normal declination angle of the microfacet.

The relationship between the macroscopic surface and microfacet is depicted in Figure 2. *N* is the normal vector of the macroscopic surface and *n* is the normal vector of the microfacet. Furthermore, *θ* is the angle between the normal vector of the microfacet and the normal vector of the macroscopic surface, i.e., the normal declination angle. As can be seen, the normal declination angle can describe the declination degree between the normal direction of microfacet and the normal direction of macroscopic surface, and its expression is as follows:

(1) θ=arccos(N⋅n)

The normal vector of the macroscopic surface is represented as *N* (*p, q,* −1) and the normal vector of the microfacet is represented as *n* (*–z_x_, –z_y_,* 1). As such, the normal declination angle can be expressed as follows:

(2) $$\theta =\arccos(\frac{1+pz_{x}+qz_{y}}{\sqrt{1+p_{}^{2}+q_{}^{2}}\sqrt{1+z_{x}{}^{2}+z_{y}{}^{2}}})$$

It can be seen that the smaller the normal declination angle of microfacet, the smaller the normal direction of the micro plane deviating from the normal direction of the macroscopic surface, causing the corresponding geometric shape to become flatter. Moreover, the larger the normal declination angle of microfacet, the greater the degree of normal declination of the microfacet from the normal direction of the macroscopic surface, causing the corresponding geometric shape to become steeper. Therefore, the normal declination angle of the microfacet can describe the geometry of the corresponding surface.

**Figure 3:**
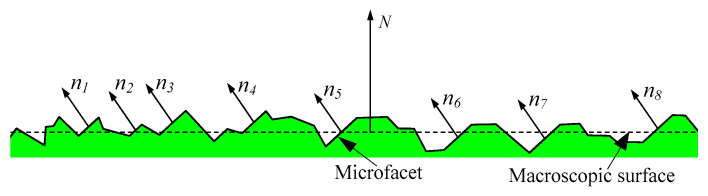
Distribution of the normal declination angle of the microfacet.

The machined surface topography is composed of numerous microfacets. Therefore, the surface topography can be described by the distribution characteristics of the normal declination angle of microfacets, as shown in Figure 3.

Based on the mathematical statistics theory, the following factors can be used to describe the distribution characteristics of the normal declination angle: centralized trend, discrete trend, skewness, and kurtosis. The arithmetic mean and standard declination are the main indexes to reflect the centralized trend and the discrete trend of the distribution of the normal declination angle [21]. The characteristic indexes are shown in Table 1.

## 3. Measurement and Analysis of the Machined Surface Topography

### 3.1. Measuring Equipment and Methods

**Figure 4:**
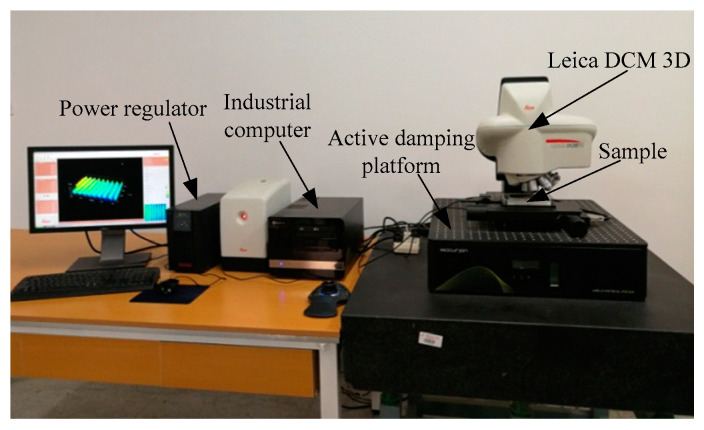
Measurement platform.

**Figure 5:**
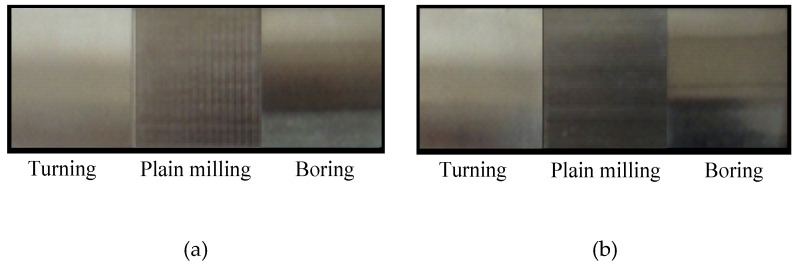
Machined sample surfaces: (**a**) *Ra* 3.2 sample surface; (**b**) *Ra* 0.8 sample surface.

Figure 4 shows the Leica DCM 3D microscope, active damping platform, industrial computer, and power regulator, which were all used to construct the measurement platform of the machined surface topography. Leica DCM 3D is a microscope that combines coaxial optical microscopy with white light interferometry. Taking the surface roughness comparison sample block produced using the Harbin Measuring Tool Factory as the measurement object, the turning, plain milling, and boring sample surface topography with *Ra* 0.8 μm and *Ra* 3.2 μm roughness grades are measured. Three roughness grade sample surfaces have obvious texture characteristics, as shown in Figure 5.

We used the Leica DCM-3D microscope with a 10× magnification lens to obtain the sample surface topography, which had a vertical resolution of less than 4 nm and a horizontal resolution of 0.56 μm. The measurement area was 1.27 mm × 0.95 mm, the sampling number was 768 points × 576 points, and the size of sampling points was 1.66 μm × 1.66 μm.

Firstly, the sample surface was properly cleaned using alcohol. Secondly, the sample’s side was clamped with tweezers and placed on a workbench. Thirdly, the moving parts of the measurement system were adjusted to determine the measurement area of the sample’s surface and to complete the focusing. Finally, the sample surface topography was measured.

During the measurement process, the system automatically adjusted the moving distance of the *z*-direction on the microscope’s scanning head, achieving the measurement of the sample surface topography according to the change of interference fringes in CMOS. A change of sample position should be avoided in the whole measurement process to ensure the accuracy of surface topography.

### 3.2. Measurement Results

**Figure 6:**
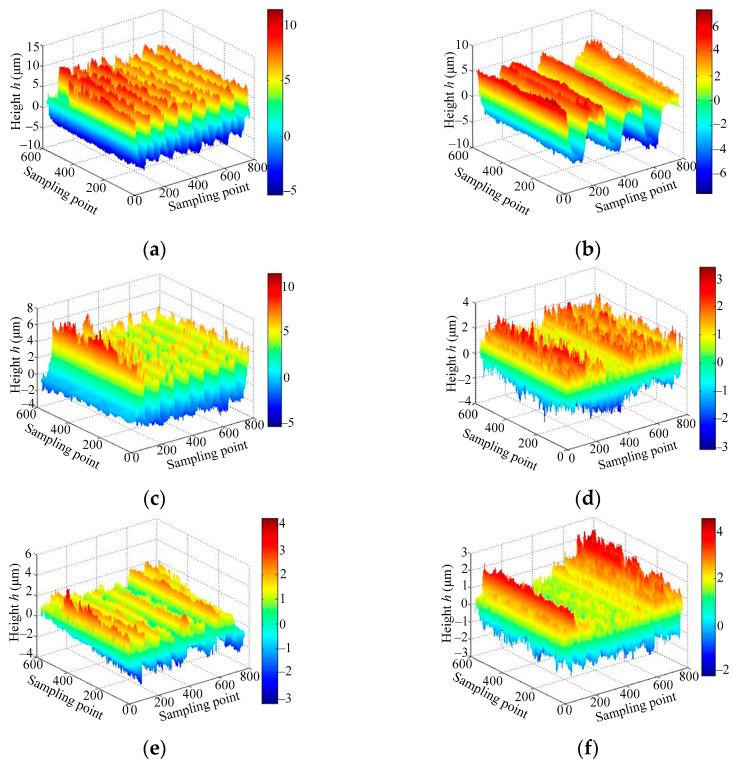
Measurement results of the sample surface topography: (**a**) *Ra* 3.2 turning; (**b**) *Ra* 3.2 plain milling; (**c**) *Ra* 3.2 boring; (**d**) *Ra* 0.8 turning; (**e**) *Ra* 0.8 plain milling; (**f**) *Ra* 3.2 boring.

Figure 6 shows the measurement results of the sample surface topography. It can be seen that the machined sample surface was smooth and flat on the macro level. However, it also showed obvious peak valley characteristics on the micro level, which belong to the non-smooth and discontinuous surface and have a typical desertification fractal structure. Under the same machined method, the peak valley distance of *Ra* 3.2 μm surfaces was larger than the *Ra* 0.8 μm surface.

### 3.3. Analysis of Machined Surface Topography

In order to study the machined surface topography by using the normal declination angle, the topography’s height data in the previous section should be processed into the normal declination angle data. Generally, a machined surface is relatively flat, and its macroscopic surface direction is perpendicular to the optical axis direction of the measurement system. Therefore, it can be assumed that the normal direction of the macroscopic surface is N (0, 0, −1). Thus, Equation (2) can be expressed as follows:

(3) $$\theta =\arccos(\frac{1}{\sqrt{1+z_{x}{}^{2}+z_{y}{}^{2}}})$$

Therefore, the surface topography height data can be processed into the normal declination angle data by using Equation (3).

**Figure 7:**
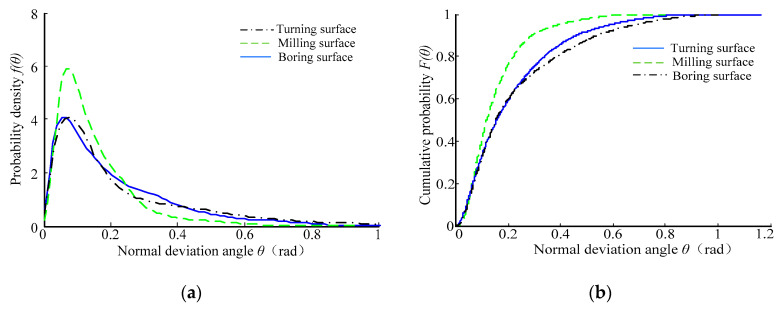
The distribution curves of the normal declination angle of the *Ra* 3.2 μm machined surface topography. (**a**) Probability density curve; (**b**) cumulative distribution curve.

Figure 7 shows the probability density curve and cumulative distribution curve of the normal declination angle of the *Ra* 3.2 μm sample surface topography. It can be seen that the probability density curves of the normal declination angle of the sample surface topography had a significant single peak feature. With the increase in the normal declination angle, the probability density function value showed a trend of “sharply increasing at first, then slowly decreasing”, and the corresponding distribution curve was steep at first and then gentle, and the tail line of the gentle curve became longer. Moreover, Figure 7b shows that the corresponding cumulative distribution function value increased when the normal declination angle also increased. The cumulative distribution curve of the plain milling surface changed steeply, followed by the turning surface and boring surface, which showed that the microfacets with small normal declination angles accounted for a large proportion on the plain milling surface.

The distribution characteristic indexes of normal deflection angles of *Ra* 3.2 μm sample surface topography are shown in Table 2.

As seen in Table 2, the arithmetic mean values of the normal declination angles for the *Ra* 3.2 μm sample surface topography were small: 0.213, 0.152, and 0.232, respectively. Moreover, the standard declination of the boring surface topography’s normal declination angle was the largest (0.207), followed by the turning surface and the plain milling surface. This indicates that normal declination angle distribution of the boring surface topography was relatively scattered, while the plain milling surface was relatively concentrated. The skewness values of the normal declination angle distribution of the *Ra* 3.2 μm sample surface topography were all greater than 0 (i.e., 1.394, 1.827, and 1.414, respectively), and the corresponding kurtosis values were 4.814, 7.176, and 4.478, respectively. Therefore, the normal declination angle distribution of the *Ra* 3.2 μm sample surface topography was characterized by right skewness and a sharp peak.

**Figure 8:**
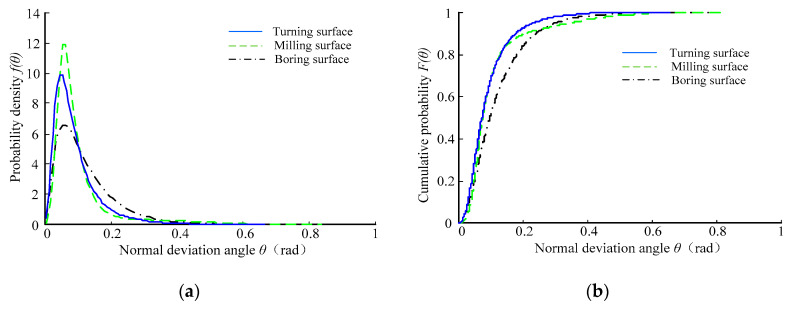
The distribution curves of the normal declination angle of *Ra* 0.8 μm machined surface topography. (**a**) Probability density curve; (**b**) cumulative distribution curve.

Figure 8 shows the probability density curve and cumulative distribution curve of the normal declination angle of the *Ra* 0.8 μm sample surface topography. It can be seen that the probability density curve of the normal declination angle of the *Ra* 0.8 μm sample surface topography also had a significant single peak feature. The probability density curve was steep at first and then gentle, and the tail line of the gentle curve was longer. Moreover, Figure 8b shows that the cumulative distribution function value increased with increasing normal declination angle of the microfacet. The change in the boring surface’s cumulative distribution curve was gentle.

Table 3 shows the distribution characteristics indexes for the normal deflection angle of the *Ra* 0.8 μm sample surface topography. 

As seen from Table 3, the arithmetic mean values of the normal declination angles of *Ra* 0.8 μm sample surface topography were small (0.12, 0.106, and 0.091, respectively). The standard declination for the boring surface topography’s normal declination angle was the smallest, with a value of 0.09, followed by the plain milling surface and turning surface, which showed that the distribution of the plain milling surface topography’s normal declination angle was relatively concentrated, while the boring surface was relatively scattered. The skewness values of the normal declination angle distribution for the *Ra* 0.8 μm sample surface topography were 1.591, 2.714, and 1.926, respectively, and the corresponding kurtosis values were 6.448, 11.152, and 8.08, respectively, indicating that the normal declination angle distribution of the *Ra* 0.8 μm sample surface topography was characterized by right skewness and a sharp peak.

**Figure 9:**
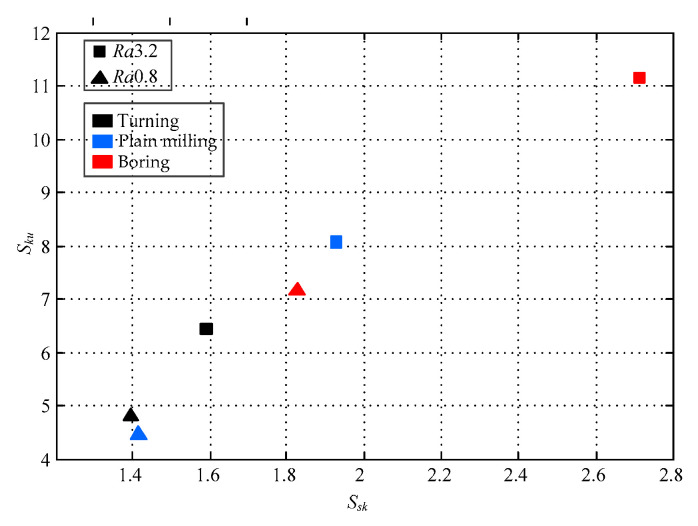
The skewness and kurtosis values for the turning, plain milling, and boring surfaces for selected roughness grade of the normal declination angle distribution.

Figure 9 shows the skewness and kurtosis values for the turning, plain milling, and boring surfaces for selected roughness grade of the normal declination angle distribution. For the *Ra* 3.2 turning, plain milling, and boring surfaces, *S_sk_* was obtained (i.e., 1.3 to 1.9). The decrease in the roughness grade, down to *Ra* 0.8, had a significant influence on surface topography characteristics. *S_sk_*, in this case, ranged from 1.5 to 2.8. It is mainly caused by the normal declination angle for most *Ra* 0.8 surface microfacets, which are relatively small. *S_ku_* values close to 12 for the *Ra* 0.8 plain milling surface prove that a steeper normal declination angle distribution can be obtained using the plain milling method.

## 4. Characterization Model of Machined Surface Topography

### 4.1. Lognormal Distribution Characterization Model

The analysis results of the machined surface topography show that the distribution characteristics of the normal declination angle of the microfacet corresponding to the surface topography are different with different machined methods. Moreover, the features of different rough surfaces are different under the same machined conditions. The smaller the surface roughness, the more concentrated the normal declination angle distribution of microfacet; the larger the surface roughness, the more dispersed the normal declination angle distribution of the microfacet. Therefore, the features of the machined surface topography are mainly related to the machined method and surface roughness.

The normal declination angle distribution of the machined surface presents significant unimodality, with right skewness and sharp peak characteristics. Based on lognormal distribution function, the normal declination angle of microfacet *θ* is taken as a random variable, and the characterization model of machined surface topography can be expressed as follows:

(4) $$f(\theta )=\frac{a}{\sqrt{2\pi }}\exp\left[-{\left(\ln\theta -b\right)}^{2}\right]$$

where *a* and *b* are the undetermined parameters. *a* reflects the kurtosis of distribution characteristics of the normal declination angle, which is related to the surface roughness; *b* reflects skewness of the distribution characteristic of the normal declination angle, which is related to the machined method.

### 4.2. Parameters of Characterization Model

The genetic algorithm (GA) was herein adopted to confirm the characterization model parameters. GA is a global searching method based on biological natural selection and natural genetic mechanism, and the global optimal solution is found in the process of evaluating multiple solutions [22]. In the process of using genetic algorithm to solve the problem, the objective function and variables of the problem should be determined first, then some individuals should be initialized, and finally the optimal solution can be found through iteration.

Therefore, the minimum mean square error between the calculation results of lognormal distribution characterization model and the distribution results of the normal declination angle are taken as the objective function.

(5) $$E(a,b)={\sum_{\theta }g(\theta )\left[f_{\mathrm{model}}(a,b)-f_{\mathrm{measured}}(\theta )\right]}^{2}\to \min$$

where *f*_model_ is the calculation results of lognormal distribution characterization model; *f*_measured_ is the distribution results of the normal declination angle; and *g*(*θ*) is a weight function.

Supposing that the object function is less than 0.01, according to the data of the normal declination angle, the parameters of the lognormal distribution characterization model can be confirmed with GA, as shown in Table 4.

### 4.3. Model Validation and Error Analysis

To verify the accuracy of the lognormal distribution characterization model, the characterization model was used to calculate the distribution of the normal declination angle. The results were compared with the normal distribution characterization model [23], Beckmann distribution characterization model [24], and exponential distribution characterization model [25].

Firstly, the normal distribution characterization model, Beckmann distribution characterization model, and exponential distribution characterization model were simplified with the normal declination angle of the micro plane as the variable. The results are shown in Table 5.

**Figure 10:**
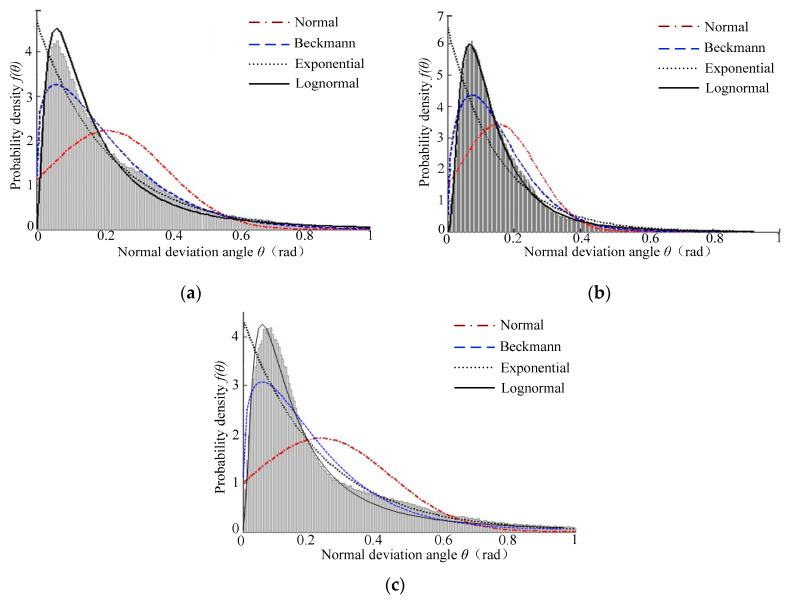
Distribution results for the normal declination angle of the *Ra* 3.2 machined surface topography: (**a**) Turning surface, (**b**) plain milling, and (**c**) boring surface.

Figure 10 shows the calculated results of each characterization model on *Ra* 3.2 surfaces, and the statistical histogram is the distribution results of the normal declination angle. It can be seen that the calculated results of the normal distribution characterization model on the distribution of the turning surfaces topography’s normal declination angle were not ideal, and there were large errors in the peak and tail of the fitting curve, which indicated that the normal distribution characterization model was not suitable for describing the characteristics of turning surface topography. The Beckmann distribution characterization model results were similar to those of the normal declination angle distribution, both of which had characteristics of a single peak and right skewness. However, the top of the model’s fitting curve was gentle, while the top of the normal declination angle distribution curve was sharp, so there was a large deviation in the fitting results. There was a large deviation between the calculated results of the exponential distribution characterization model and the normal declination angle distribution, which cannot describe the characteristics of the turning surface topography. The calculated results of lognormal distribution characterization model were similar to those of the normal declination angle distribution, both of which had the characteristics of a single peak and right skewness. The curve peak of the *Ra* 3.2 turning surface was sharper than the normal declination distribution. This was mainly due to the dispersed distribution of the normal declination angle of the *Ra* 3.2 turning surface. The lognormal distribution characterization model ensured the accuracy of the whole calculation results, which led to local errors.

**Figure 11:**
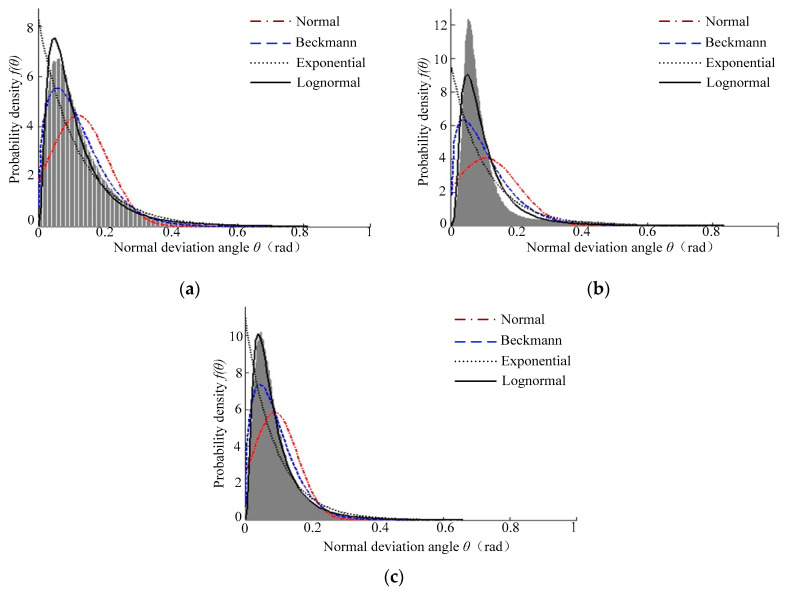
Distribution results for the normal declination angle of the *Ra* 0.8 machined surface topography: (**a**) turning surface, (**b**) plain milling, and (**c**) boring surface.

Figure 11 shows the results of each characterization model on *Ra* 0.8 surfaces, wherein the statistical histogram is the distribution results of the normal declination angle. It can be seen that the calculated results for the normal distribution characterization model were not ideal. The top of the distribution curve for the Beckmann distribution characterization model was gentle, however the calculation results were not good for a small normal declination angle—the error was large. There was a large deviation between the calculation results of the exponential distribution characterization model and the distribution of the normal deflection angle. The calculated results of the lognormal distribution and distribution of the normal deflection angle had the characteristics of a single peak and right skewness. The peak state was basically the same, so the overall calculation effect was considered to be good.

The following two parameters were selected to analyze error:

(1) Mean error (*M_E_*):

(6) $$M_{E}=\left|{\bar{f}}_{1}-{\bar{f}}_{2}\right|$$

(2) Root mean square error (RMSE), which can reflect the deviation degree of the calculation results for the lognormal distribution characterization model and the distribution results of the normal declination angle.

(7) $$\mathrm{RMSE}=\sqrt{\frac{1}{n}{\sum_{i=1}^{n}\left(f_{1i}-f_{2i}\right)}^{2}}$$

Here, *i* is the sampling point number of the normal declination angle, and *i* = 1,2, …*n*. *f_1i_* is the calculated result of the characterization model. *f_2i_* is the distribution result of the normal declination angle.

Table 6 shows the errors between the calculated results and the distribution results. For the *Ra* 3.2 sample surface, the mean error and root mean square error of the turning surface were the largest: 0.224 and 0.024, respectively. For the *Ra* 0.8 sample surface, the mean error and root mean square error of the plain milling surface were the largest: 0.264 and 0.045, respectively. The error between the calculation results of the lognormal distribution characterization model and the normal declination angle was small. Therefore, the lognormal distribution characterization model accurately described the machined surface topography.

## 5. Conclusions

In this article, a novel characterization method—the microfacet-based surface topography normal declination angle—was proposed for machined surface topography. The normal declination angles of the measured surface topography were calculated and analyzed. The probability density curve of the normal deflection angle of the machined surface had a single, right-skewing peak, which related with the machined method and surface roughness. Moreover, the lognormal distribution characterization model was established. An error analysis of the characterization model was conducted and the maximal RMSE error was 4.5%. Compared with other characterization models, the present lognormal distribution characterization model achieved better description capability for the machined surface topography.

The proposed characterization method can serve as an effective and novel way to describe machined surface topography characteristics, especially surface topography with significant textural features. It can be utilized in the reconstruction of machined surfaces and the prediction of functional behaviors, such as contact, wear, or friction of engineering assemblies manufactured via machining. However, due to the limitation of our research conditions, the proposed method was not applied to non-metallic material surfaces, such as monocrystalline silicon. In the future, we will test more surfaces and further optimize our model to enhance accuracy.

## Figures and Tables

**Table 1:** The adopted distribution characteristic index.

General Outlines for the Adopted Characteristic Index	The Normal Declination Angle of the Microfacets Is Denoted by *θ_i_*, Where *i* = 1, 2, …*n*.
Arithmetic average (*μ*)	$\mu =\frac{1}{n}\sum_{i=1}^{n}\theta_{i}$
Standard declination (*σ*)	$\sigma =\sqrt{\frac{1}{n}\sum_{i=1}^{n}{\left(\theta_{i}-\mu \right)}^{2}{}_{}}$
Skewness (*S*)	$S=\frac{1}{n}\sum_{i=1}^{n}{\frac{{\left(\theta_{i}-\mu \right)}^{3}}{\sigma^{3}}}_{}$ When *S* = 0, the probability density curve is normal. When *S* < 0, the probability density curve is left-skewed. When *S* > 0, the probability density curve is right-skewed.
Kurtosis (*K*)	$K=\frac{1}{n}\sum_{i=1}^{n}{\frac{{\left(\theta_{i}-\mu \right)}^{4}}{\sigma^{4}}}_{}$ When *K* < 3, the normal skew angle distribution curve presents a low peak state; when *K* > 3, the normal skew angle distribution curve presents a sharp peak feature.

**Table 2:** Distribution characteristic index of the normal declination angle of *Ra* 3.2 μm sample surface topography.

Surface Type	Arithmetic Average	Standard Declination	Skewness		Kurtosis
Turning	0.213	0.179		1.394	4.814
Plain milling	0.152	0.116		1.827	7.176
Boring	0.232	0.207		1.414	4.474

**Table 3:** Distribution characteristic index of the normal declination angle of *Ra* 0.8 μm sample surface topography.

Surface Type	Arithmetic Average	Standard Declination	Skewness	Kurtosis
Turning	0.12	0.089	1.591	6.448
Plain milling	0.106	0.068	2.714	11.152
Boring	0.091	0.090	1.926	8.08

**Table 4:** Parameters of the lognormal distribution characterization model.

Surface Type	*Ra* 3.2		*Ra* 0.8	
	*a*	*b*	*a*	*b*
Turning	1.06	−1.92	1.21	−2.41
Plain milling	1.33	−2.14	1.42	−2.52
Boring	1.36	−2.65	1.57	−2.71

**Table 5:** Parameters of the lognormal distribution characterization model.

Characterization Model	Normal Distribution	Beckmann Distribution	Exponential Distribution	Lognormal Distribution
Expression	12πσe−(θ−μ)2σ2	1σ2cos4θe−tg2θσ2	12πσ2e−θσ2	$\frac{a}{\sqrt{2\pi }}e^{-{\left(ln\theta -b\right)}^{2}}$

**Table 6:** Error results.

Roughness	Surface Type	*M_E_*	*RMSE*
*Ra* 3.2	Turning	0.224	0.024
	Plain milling	0.112	0.009
	Boring	0.209	0.021
*Ra* 0.8	Turning	0.217	0.021
	Plain milling	0.264	0.045
	Boring	0.106	0.006
